# Trait-Related Impulsivity, Affective Temperaments and Mood Disorders: Results from a Real-World Multicentric Study

**DOI:** 10.3390/brainsci12111554

**Published:** 2022-11-15

**Authors:** Mario Luciano, Gaia Sampogna, Emiliana Mancuso, Alessio Simonetti, Pasquale De Fazio, Marco Di Nicola, Giorgio Di Lorenzo, Maria Pepe, Fabio Sambataro, Maria Salvina Signorelli, Alexia Emilia Koukopoulos, Roberto Delle Chiaie, Gabriele Sani, Andrea Fiorillo

**Affiliations:** 1 Department of Psychiatry, University of Campania Luigi Vanvitelli, 80138 Naples, Italy; 2 Department of Neuroscience, Section of Psychiatry, Università Cattolica del Sacro Cuore, 00168 Rome, Italy; 3 Department of Psychiatry, Fondazione Policlinico Universitario Agostino Gemelli IRCCS, 00168 Rome, Italy; 4 Department of Health Sciences, University Magna Graecia of Catanzaro, 88100 Catanzaro, Italy; 5 Department of Systems Medicine, University of Rome Tor Vergata, 00133 Rome, Italy; 6 Department of Neuroscience, University of Padova, 35121 Padua, Italy; 7 Psychiatry Unit, Department of Clinical and Experimental Medicine, University of Catania, 95123 Catania, Italy; 8 Department of Human Neurosciences, Sapienza University, 00161 Rome, Italy

**Keywords:** mood, bipolar, major depression, impulsivity, temperaments, BIS-11, TEMPS, outcome, suicidality, seasonality

## Abstract

Trait-related impulsiveness is highly prevalent in patients with mood disorders, being associated with negative outcomes. The predictive role of affective temperaments on trait-related impulsivity is still understudied. The aim of the present study is to investigate the relationship between impulsivity and affective temperaments in a sample of euthymic patients with mood disorders. This is a real-world multicentric observational study, carried out at the outpatient units of seven university sites in Italy. All patients filled in the short version of Munster Temperament Evaluation of the Memphis, Pisa, Paris and San Diego and the Barratt Impulsiveness Scale. The study sample included 653 participants, mainly female (58.2%), with a mean age of 46.9 (±14.1). Regression analyses showed that higher levels of trait-related impulsivity were associated to suicide attempts (*p* < 0.000), the presence of psychotic symptoms during acute phases (*p* < 0.05), a seasonal pattern (*p* < 0.05), a lower age at onset of the disorder (*p* < 0.05), cyclothymic (*p* < 0.01) and irritable temperaments (*p* < 0.01). The results of our study highlight the importance to screen patients with mood disorders for impulsivity and affective temperaments in order to identify patients who are more likely to present a worse outcome and to develop personalized and integrated early pharmacological and psychosocial treatment plans. Novelties of the present paper include the recruitment of patients in a stable phase, which reduced possible bias in patients’ self-reports, and the multicentric nature of the study, resulting in the recruitment of a large sample of patients with mood disorders, geographically distributed across Italy, thus improving the generalizability of study results.

## 1. Introduction 

Impulsivity, a multidimensional construct defined as the predisposition toward fast and unplanned reactions to internal and external stimuli regardless of the associated negative consequences [1], includes risk taking, disinhibition and reduced cognitive control [2]. Impulsive actions are often poorly conceived, premature, and may lead to undesired outcomes; they include two components, one is acting without inhibitions and the other is the preference for immediate pleasure over long-term planning. As a consequence, impulsive patients are reluctant to conform to contextual demands, with a consequent high prevalence of risky behaviors [3,4]. 

The Diagnostic and Statistical Manual of Mental Disorders (DSM) [5,6] lists impulsivity among criteria for mania as “an excessive involvement in pleasurable activities that have a high potential for painful consequences”. Impulsivity is a psychiatric symptom more prevalent in some mental disorders, including affective disorders, accounting for both a trait and a state dependent characteristic [7] in manic, depressive, and euthymic phases [8,9]. In particular, it has been reported that impulsivity in patients with affective unipolar and bipolar disorders is a trait-related characteristic, poorly related to the presence of active symptoms of the underlying disorder, and that has to be considered a stable aspect of the patient’s usual reactions [10]. In fact, when compared with healthy controls, patients with unipolar and bipolar disorders score significantly higher at all dimensions of impulsivity, especially in the absence of any other clinically significant symptom [7]. Impulsivity usually predicts a worse outcome, being associated with higher rates of aggressive behaviors [11], suicidality [12], comorbidity with substance use disorder [13,14] and gambling disorder [15]. In particular, as regards the association between impulsivity and suicide, impulsivity is associated, both in BD and MDD patients, with a higher number of suicide attempts and a higher lethality of suicidal acts compared to non-impulsive patients [16,17]. 

Moreover, impulsivity predicts illness severity in patients with mood disorders [18] and has a significant impact on patients’ quality of life and comorbidity with other psychiatric and physical diseases [19,20,21,22]. Additionally, the presence of impulsivity worsens sleep quality [23] and increases nonadherence to treatment [24]. 

Despite its importance in clinical settings, impulsivity has been poorly studied in patients with affective disorders, and an exhaustive clinical characterization of impulsive patients with affective disorders is not yet available. Many authors [25,26,27,28] have challenged the unipolar-bipolar dichotomy suggesting a broader conceptualization of bipolarity, echoing the manic-depressive continuum envisaged by Kraepelin [29,30]. In particular, mood disorders would be part of the so-called “broad bipolar spectrum”, where several psychopathological domains, personality and affective temperaments shape multiple and complex clinical phenotypes [27]. To address this diagnostic conundrum, the temperament concept has been revised, starting from the Kraepelinian concept of “fundamental states” [31,32]. Affective temperaments are conceptualized as a biologically determined and hereditable core of personality [2], and therefore intended as a stable trait across the lifespan. Affective temperaments, which include hyperthymic, cyclothymic, irritable, anxious and depressive states, describe the basic level of individuals’ reactivity to internal and external stimuli, impulsiveness, mood and energy. The temperamental states might be predictive of the diagnosis, symptom presentation, and the clinical features of mood disorders [26,33]. 

Despite the growing interest in affective temperaments, only a few studies have investigated the relationship between impulsivity and affective temperaments in patients with affective disorders. Specific temperamental profiles are related to impulsive behaviors in affective disorders, including compulsive buying [34], suicidality [35], aggressive behaviors [36] and criminal history [37]. 

Some studies found that cyclothymic, anxious, and irritable temperamental dispositions might represent the underlying characteristic of impulsivity [38,39]. In terms of other psychiatric disorders, affective temperaments might be important mediators of the relationship between impulsivity and illness severity in patients with binge eating disorder [40], pathological gambling [2] and panic disorder-agoraphobia [39]. However, the intertwinement between affective temperaments and impulsivity in patients with affective disorders has been poorly studied, although the importance to understand their relationship is largely acknowledged.

Available studies on the association between impulsivity and the clinical indicators of patients with mood disorders present several weaknesses. In fact, the majority of studies have been carried out with small sample sizes, mostly recruited in single centers. Moreover, only rarely have patients been recruited when in a stable phase of the disorder; that is one of the major flows in available literature, since patients’ self-reports about trait-related impulsivity and about affective temperaments could be significantly influenced by the presence of active symptoms, both in the depressive and in the manic/hypomanic phases.

Based on these premises, in this study we aim to investigate, in a multicentric observational study, the relationship between impulsivity and affective temperaments in a sample of euthymic patients with bipolar I disorder, bipolar II disorder, cyclothymic disorder and major depressive disorder. We hypothesized that: (1) patients with trait-related impulsivity presents a more severe course of the disorder, characterized by a higher number of relapses and hospitalizations as well as the presence of suicidality and seasonal pattern; and (2) affective temperaments characterized by emotional instability (i.e., cyclothymic and irritable) are associated to the presence of trait-related impulsivity. 

## 2. Methods

This is a real-world multicentric observational study, carried out at the outpatient units of seven university sites in Italy (University of Campania “L. Vanvitelli”, University of Catania, University Magna Graecia of Catanzaro, University Cattolica del Sacro Cuore, University of Padova, Sapienza University of Rome and University of Rome Tor Vergata). 

All patients referred to their unit between November 2020 and January 2021 were included in the study, if they met the following inclusion criteria: (1) clinical diagnosis of type-I or type-II bipolar disorder, cyclothymic disorder or major depressive disorder; (2) age between 18 and 65 years; (3) willingness to participate in the study, expressed by written informed consent provided upon complete description of the protocol; (4) being in a stable phase of the disorder; and (5) receiving treatment according to the NICE guidelines for major depression [41] or bipolar disorder [42]. 

Eligible patients were provided with all relevant information on the study characteristics in order to collect informed consent. Subsequently, a diagnostic interview was run through the Structured Clinical Interview for DSM-5 disorders in order to confirm the diagnosis. A second appointment was arranged in order to administer all assessment instruments included in the study. All patients have been recruited on a voluntary basis. No incentives have been provided to patients.

Patients were excluded in the case of: (1) inability to give a written consent to participate in the study; (2) diagnosis of any neurological disease; and (3) the presence of actual drug and/or alcohol abuse. The study was carried out in accordance with the latest version of the Declaration of Helsinki and was approved by the Local Research Ethic Committee of the coordinator center (Protocol number ID 5016).

### 2.1. Procedures and Measures

#### 2.1.1. Socio-Demographics Characteristics

Socio-demographic (i.e., gender, age at study entry, work, and educational level) and clinical characteristics (i.e., age at onset, lifetime number of affective episodes and of hospitalizations, suicide attempt seasonality, the presence of psychotic symptoms during affective episodes) were collected with an ad-hoc schedule. Previous mood episodes have been defined according to the DSM-5 criteria. 

#### 2.1.2. Psychopathological Assessments 

Since current depressive and manic or hypomanic episodes influence the assessment of trait-impulsivity (Powers et al., 2013), patients not in a stable phase for at least one month previously were not enrolled in the study. Euthymic phases were defined as a score ≤ 9 at the Hamilton Rating Scale for Depression [43] and a score ≤ 11 at the Young Mania Rating Scale [44], and not fulfilling the criteria for a current affective episode according to DSM-5 criteria. 

The Italian version of the Barratt Impulsiveness Scale (BIS-11) was used to assess trait-related impulsivity [45]. Each BIS-11 item is rated on a 4-point Likert scale (from 1 = rarely to 4 = almost always/always). The total summed score ranges from 30 to 120 points, with higher values indicating higher impulsivity traits. The BIS-11 internal consistency for the Italian translation was satisfactory (Cronbach’s α = 5.79). The 2-month retest reliability of BIS-11 total score was also acceptable (r = 5.889, *p* < 0.001) [45]. According to Standford et al. [46], a score ≥ 72 indicates the presence of impulsiveness as a trait-related dimension. The BIS-11 also allows the identification of three second-ordered factors: (1) motor impulsiveness (characterized by a deranged and unplanned response to stimuli); (2) non-planning impulsiveness (characterized by a lack of looking forward or forethought); and (3) attentional impulsiveness (identified by an individual’s inability to concentrate on cognitively complex situations) [18]. 

Affective dispositions were assessed with the Italian short-version of the Munster Temperament Evaluation of the Memphis, Pisa, Paris and San Diego (bTEMPS-M), a 35-item self-administered questionnaire, which allows the identification of the five affective dispositions (cyclothymic, depressive, hyperthymic, irritable and anxious), according to Akiskal classification [47]. Cronbach’s alpha coefficients of subscales of the Italian version of the bTEMPS-M ranged from 0.808 to 0.898, and Kaiser Meyer-Olkin (KMO) measure of sampling adequacy for the sample was 0.914, corresponding to the recommended value of at least 0.6; Bartlett’s Test of Sphericity was statistically significant (*p* < 0.0001), supporting the factorability of the correlation matrix [47].

#### 2.1.3. Statistical Analyses

Descriptive statistics were calculated for baseline characteristics, along with scores of included assessment instruments. Data were presented as means (M) and standard deviations (SD), or as frequencies and percentages (%). Kolmogorov–Smirnov test was used to verify the normal distribution of data. 

According to the BIS-11 total score (≥72), the global sample was divided in impulsive and non-impulsive groups (IG and NIG groups, respectively). Differences between groups were calculated with Student’s t-test or χ^2^, as appropriate. Moreover, χ^2^ test was used to assess the association between affective temperaments and the presence of impulsiveness. All patients who had a total score ≥ 72 at the BIS-11 were further divided according to the prevalent impulsive dimension (i.e., motor, non-planning and attentional impulsiveness). Kendall’s rank correlation analysis was used to assess the association between affective temperaments, the presence of impulsiveness and prevalent impulsive dimensions. Two series of logistic regression analyses were performed, with the presence of impulsivity and each prevalent impulsive dimension, including affective dispositions, as independent variables. In model one, the dependent variables, selected among those who were statistically significant at the univariate analyses, were diagnosis, age at onset, number of affective episodes and of involuntary hospitalizations, suicide attempts, seasonality and the presence of psychotic symptoms during affective episodes. In model two, the four affective temperaments, which resulted statistically significant at univariate analyses, were added to the dependent variables already included in model one. Missing data have been handled using the multiple imputation approach [48,49]. Statistical significance was set for *p* < 0.05. Statistical analyses were performed through the Statistical Package for Social Sciences, version 21.

## 3. Results

### 3.1. Main Socio-Demographic and Clinical Characteristics of the Total Sample

The study sample included 653 participants, mainly affected by bipolar disorder (55.7%), major depressive disorder (35.8%) or cyclothymic disorder (8.4%). Patients were mainly female (58.2%), with a mean age of 46.9 (±14.1) years and an educational level of 13.3 (±3.6) years. They had a mean age at the onset of the disorder of 31.3 (±13.1) years. Thirty-nine percent of patients were living with a partner and 54.7% were employed. In all, 17.9% of patients committed suicide attempts, and 32.5% had a seasonal pattern of the disorder. The number of affective episodes was 4.5 ± 5.0, whereas the mean number of voluntary and involuntary hospitalizations was 2.4 ± 2.5 and 1.5 ± 0.9, respectively. A total of 29.5% of patients reported the presence of psychotic symptoms during acute phases (Table 1). In total, 356 patients reported a score of ≥72 at BIS-11, and were included in the group with impulsivity.

### 3.2. Differences among Patients with and without Impulsivity

Patients in the impulsive group (IG) were more often females (56.2%), with a mean age of 45.2 ± 13.7 and an educational level of 13.3 ± 3.5. Patients in the non-impulsive group (NIG) were mostly females (61.1%), with a mean age of 48.9 ± 14.1 years (Table 2). 

A significantly higher proportion of patients with bipolar disorder reported the presence of trait-related impulsiveness (62.4% in the IG vs. 37.6% in the NIG group, *p* < 0.0001), while impulsivity was less frequently reported by patients with major depression (43.6% in the IG vs. 56.4% in the NIG group, *p* < 0.01). 

Moreover, compared to patients from the NIG, IG patients reported a lower age at onset of the disorder (29.4 ± 12.1 vs. 33.5 ± 13.8 years, *p* < 0.0001), a higher number of affective episodes (5.1 ± 5.5 vs. 3.9 ± 4.3, *p* < 0.0001), of suicide attempts (27.5% vs. 6.7%, *p* < 0.0001), of involuntary hospitalizations (1.6 ± 1.1 vs. 1.2 ± 0.5, *p* < 0.05) and of psychotic symptoms during affective episodes (37.1% vs. 20.8%, *p* < 0.0001). Finally, the seasonal pattern was significantly more frequent in IG participants (37.6% vs. 26.2%, *p* < 0.001). 

### 3.3. Impulsivity and Affective Temperaments

**Figure 1:**
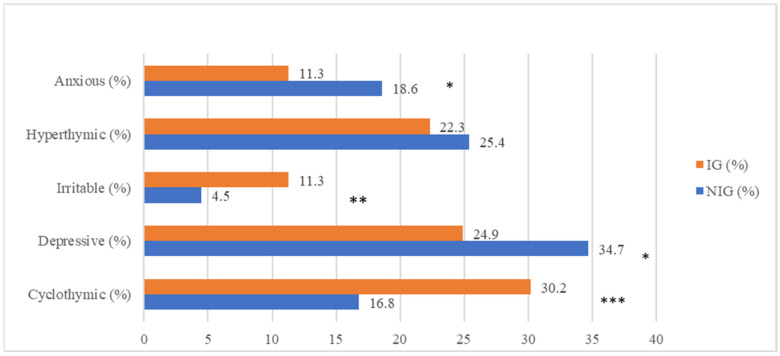
Distribution of trait-related impulsivity among affective temperaments. * *p* < 0.01; ** *p* < 0.001; *** *p* < 0.000, NIG: non impulsivity group; IG = impulsivity group.

In the total sample, most participants showed a depressive temperamental disposition (28.9%), followed by cyclothymic (23.9%), hyperthymic (23.4%), anxious (14.4%) and irritable (8.1%) temperaments. Cyclothymic (30.2% vs. 16.8%, *p* < 0.0001) and irritable (11.3% vs. 4.5%, *p* < 0.001) temperamental dispositions were more frequently associated with impulsivity. On the contrary, the number of impulsive patients was significantly lower in the depressive (24.9% vs. 34.7%, *p* < 0.01) and anxious temperament groups (11.3% vs. 18.6%, *p* < 0.01) (Figure 1).

Correlation analyses found that cyclothymic and irritable temperaments positively correlated with the presence of trait-impulsiveness (*p* < 0.0001 and *p* < 0.01, respectively), while depressive and anxious temperaments negatively correlated with the presence of trait-impulsiveness (*p* < 0.01). Moreover, depressive temperament was negatively correlated with motor impulsiveness (*p* < 0.01) and positively correlated with non-planning impulsiveness (*p* < 0.001) (Table 3).

### 3.4. Logistic Regression Analyses

Data obtained with the logistic regression model 1 showed that higher impulsivity levels were associated with: (1) the diagnosis of bipolar disorders (OR: 1.16; 95% CI: 0.62 to 2.18; *p* < 0.01); (2) suicide attempts (OR: 4.21; 95% CI: 2.56 to 6.98; *p* < 0.000); (3) seasonal pattern (OR: 1.44; 95% CI: 0.99 to 2.09; *p* < 0.05); (4) the presence of psychotic symptoms during acute phases (OR: 1.56; 95% CI: 1.03 to 2.35; *p* < 0.05); and (5) lower age at onset of the disorder (OR: 0.98; 95% CI: 0.97 to 0.99, *p* < 0.05). Moreover, patients with a diagnosis of major depression were less likely to report impulsivity (OR: 0.8; 95% CI: 0.43 to 1.50; *p* < 0.05). 

When affective temperaments where included in the model (Model 2), the statistical significance persisted for the following variables: (1) suicide attempts (OR: 3.99; 95% CI: 2.41 to 6.63; *p* < 0.63; *p* < 0.000); (2) the presence of psychotic symptoms during acute phases (OR: 1.57; 95% CI: 1.03 to 2.09; *p* < 0.05); (3) seasonal pattern (OR: 1.43; 95% CI: 0.98 to 2.09; *p* < 0.05); and (4) lower age at onset of the disorder (OR: 0.98; 95% CI: 0.97 to 0.99, *p* < 0.05), while the variable “diagnosis” was no longer statistically significant. With regards to affective temperaments, patients with cyclothymic (OR: 2.12; 95% CI: 1.29 to 3.49; *p* < 0.01) and irritable temperaments (OR: 3.12; 95% CI: 1.48 to 6.52; *p* < 0.01) were more likely to present trait-related impulsivity (Table 4). 

As regards the dimensions of impulsiveness, motor aggressiveness was associated with the diagnosis of MDD at the regression analyses (Model 1, OR: 0.32, 95% CI: 0.13 to 0.76, *p* < 0.05; Model 2, OR: 0.36, 95% CI: 0.15 to 0.89, *p* < 0.05). Moreover, patients with a higher number of affective episodes were more likely to present motor aggressiveness both in Model 1 (OR: 1.07; 95% CI: 1.02 to 1.13; *p* < 0.05) and in Model 2 (OR: 1.07; 95% CI: 1.02 to 1.13; *p* < 0.05) (Table 5).

## 4. Discussion

To our knowledge, this is the first study on the clinical characterization of a large sample of patients diagnosed with unipolar or bipolar affective disorder presenting a trait-related impulsivity in real-world settings. Moreover, we have investigated the relationship between the different affective dispositions and the development of trait-related impulsivity. 

One of the most striking findings of our study is the association of impulsivity with different course indicators, including age at onset of the disorder, suicidality, seasonality, and the presence of psychotic symptoms during affective episodes, which can all be considered prognostic factors of worse outcome. This finding would imply that the long-term outcome of unipolar and bipolar disorders is heavily influenced by trait-related impulsivity, independent from the specific mood disorder patients are suffering from. This finding is in line with previous evidence that impulsivity is strongly related to illness severity in patients with affective disorders [18]. 

In fact, in bipolar disorder, impulsivity is associated with earlier onset, more frequent suicidal behaviors and relapses [12,16], reduced time in euthymic phase [50], rapid cycling [51] and more frequent substance use or comorbid behavioral addictions [52,53]. However, while several studies have been carried out on the association between impulsivity and BD, little is known on the relationship between impulsivity and MDD. The little available evidence showed that impulsivity can also worsen the long-term outcome in MDD, by increasing suicidality [54], substance misuse and mood instability [7]. In particular, impulsivity has been found to be a strong predictor of future suicidal attempts in a mixed sample of patients with BD and MDD, without differences between the two affective disorders [16]. 

Zhang et al. [55] reported that impulsiveness could act as a moderator in influencing suicidal ideation, and in precipitating the onset of depressive symptoms, which are considered an important bridge between impulsivity and suicidal ideation. Along these lines, we can anticipate that in our sample impulsivity significantly influenced mood instability and patients’ ability to successfully cope with stressful life events. Accordingly, impulsivity may account for a distinct phenotype of affective disorders, characterized by rapid and unplanned reactions, failure in understanding the negative consequences of those reactions, attentional bias, rigid cognitive style and reduced coping strategies [35]. This interpretation is in line with the evidence that trait-related impulsivity is linked with biological aspects [56,57] including gene expression [58,59], alterations in functional brain connectivity and the reduced availability of serotonin transporter in the anterior cingulate cortex [60]. 

Therefore, affective disorders with trait-related impulsivity might be considered a distinct subgroup, characterized by higher mood fluctuations, greater symptom severity and worse outcome. From a clinical viewpoint, the recognition that impulsivity traits are a marker of worse long-term outcome in patients with affective disorders might be helpful for identifying those patients who are at higher risk of relapses and of suicide attempts since the beginning of the disorder [61]. Moreover, characterizing affective patients based on the presence of impulsivity might guide clinicians in choosing the most appropriate pharmacological and psychosocial intervention with a documented effect in improving cognitive styles and behavioral skills. 

Furthermore, the similar distribution of trait-related impulsivity in both bipolar and unipolar patients is noteworthy. Although we found a higher recurrence of trait-related impulsiveness in BD, the impact of the diagnosis on the likelihood to present trait-related impulsivity is relevant only when affective temperaments are not considered. MDD seems to have a protective role against impulsivity only with respect to motor impulsiveness [62]. Thus, the question whether impulsivity should represent a common trait in bipolar and unipolar disorders is still ongoing and available evidence is not robust enough to give a definite answer. Our data confirm that impulsivity is relatively independent from mood states and the diagnosis of mood disorder, as also shown by Henna et al. and Peluso et al. [7,62]. 

With respect to the second research aim (i.e., assessing the relationship between affective temperaments and impulsivity), we found that cyclothymic and irritable temperaments were strongly correlated with trait-related impulsivity, even after controlling for several sociodemographic and clinical factors. Interestingly, the presence of these two affective dispositions was even more strongly associated with impulsivity than the diagnosis itself, suggesting that the diagnostic categories, as conceived by current diagnostic manuals, seem not to catch the complexity of affective disorders [63,64].

This result supports the notion that mood disorders belong to a broad affective spectrum, where individual temperaments, impulsivity traits, personality traits and psychopathological characteristics merge in multiple complex clinical phenotypes [27]. These characteristics might guide clinicians toward a better clinical characterization and personalized treatment plan of patients [65,66,67], suggesting that the trans-diagnostic approach to mental disorders seems more useful for clinical practice than the categorical classification [68,69,70]. 

Only the hyperthymic temperament did not show any correlation with trait-related impulsivity and its sub-dimensions. Consistently with available literature, our finding shows that the hyperthymic temperament is orthogonally different from all other affective dispositions [71] and is mainly characterized by mood-emotional intensity and high levels of energy. Incidentally, the other four temperaments can be grouped into a single disposition, mainly characterized by emotional instability and rapid mood fluctuations.

However, affective temperaments have been only rarely linked to impulsivity. In fact, a very few studies have explored the possible association between affective dispositions and trait-related impulsivity and its dimensions. To our knowledge, the only study which has directly assessed the relationship between affective temperaments and impulsivity was carried out in a non-clinical sample and found that cyclothymic disposition was strongly correlated with high levels of impulsivity and pathological gambling [2]. The lack of evidence on this association is surprising, since some affective dispositions, such as irritable and cyclothymic temperaments, and trait-related impulsivity, have several elements in common, including mood instability and reactivity [71], reduced cognitive performance under stressful situations, a higher recurrence of aggressive behaviors and substance abuse [36], and emotional dysregulation [72,73], with important therapeutic and prognostic implications. These common characteristics were confirmed by our finding of the association of cyclothymic and irritable temperaments with trait-related impulsivity. 

One of the main strengths of the present study is the fact that only patients in a stable phase of the disorder have been recruited, since the levels of impulsivity are influenced by the presence of several affective symptoms, especially in acute depressive and manic phases of bipolar disorder. We believe that this methodological choice adds value to our findings, since most available evidence had been collected in patients regardless of the phase of the disorder. In fact, previous studies did not clarify whether differences in impulsivity levels found across clinical states were mainly due to the clinical characteristics of subjects (i.e., with predominately depressive or manic phases of bipolar disorder) or to the patients’ temperamental predispositions. Moreover, the multicentric design of our study, the large sample size and the naturalistic settings in which patients have been recruited, make our results generalizable to patients with affective disorders usually seen in routine clinical settings. 

Our results, although very significant from a clinical viewpoint, should be considered in the light of some limitations. First, the cross-sectional design of the study does not allow any speculation regarding a cause-effect relationship. Longitudinal studies might better ascertain whether the stable component of impulsivity in affective disorders is a consequence of repeated mood episodes or is a trait-characteristic preceding the onset of mood disorder. Second, the correlation between impulsivity, affective temperaments, clinical variables and other psychiatric comorbidities, was not considered. However, we will analyze all possible comorbidities in subsequent analyses.

Moreover, we decided to analyze BIS-11 using cut-off scores, rather than adopting continuous measures. Despite the dichotomization of continuous variables in regression analyses, which may have reduced their statistical power [74], this methodological choice was due to the fact that validated BIS-11 cut-off are available [46], and to the need to identify clinical correlates of patients with trait-related impulsivity, which was not possible with a continuous variable. Lastly, both affective temperaments and impulsivity have been assessed with self-reported measures. However, the TEMPS and BIS-11 are among the most adopted instruments to assess affective disposition and impulsivity in clinical samples, allowing us direct comparisons with other available studies.

## 5. Conclusions

Our study represents a first attempt to clinically characterize patients with high impulsivity traits and helps to understand the relationship between impulsivity and affective temperaments, highlighting that impulsivity might be related to a worse outcome profile both in unipolar and bipolar disorders. Innovations coming from this study include the fact that only patients in a stable phase of mood disorders have been recruited, avoiding possible bias in patients’ reports on trait-related impulsivity and affective dispositions. Additionally, the multicentric nature of the study allowed the recruitment of a large sample of patients with mood disorders, geographically distributed across Italy, thus improving the generalizability of our findings.

Further research on the clinical correlates of impulsivity should include: (1) a control group of individuals without any major mental disorders, in order to assess whether the association between impulsivity and affective temperaments precede the onset of a mental disorder; (2) a longitudinal assessment of impulsivity in order to verify causal relationships among the two dimensions; and (3) the recruitment of patients with a diagnoses other than those belonging to mood disorders, in order to verify the hypotheses that the relationship between impulsivity and affective dispositions is transdiagnostic. 

The clinical interpretation of our findings is that patients with affective episodes should be screened for impulsivity and for affective temperaments in order to identify as early as possible those patients who are more likely to present a more severe course and worse outcome. Such an early screening might be useful to develop personalized and integrated early pharmacological and psychosocial treatment plans to patients with affective disorder, according to the precision medicine approach.

## Figures and Tables

**Table 1:** Sociodemographic and clinical characteristics of the total sample.

	Total Sample (*n* = 653)
Age (M ± DS)	46.9 ± 14.1
Gender, M, % (n)	41.8 (275)
Living situation, with partner yes % (*n*)	39 (256)
Years of education (M ± DS)	13.3 ± 3.6
Employed, yes, % (*n*)	54.7 (360)
Age at onset (M ± DS)	31.3 ± 13.1
Diagnosis, % (n)	
Bipolar disorder	55.7 (364)
Major depression	35.8 (234)
Cyclothymic disorder	8.4 (55)
Number of affective episodes (M ± DS)	4.5 ± 5.0
Number of hospitalizations (M ± DS)	2.4 ± 2.5
Number of involuntary hospitalizations (M ± DS)	1.5 ± 0.9
Suicide attempts, yes, % (*n*)	17.9 (118)
Seasonality, yes, % (*n*)	32.5 (212)
Presence of psychotic symptoms during acute phases, yes, % (*n*)	29.5 (195)
Trait-related impulsivity (BIS-11 total score ≥ 72), yes, % (*n*)	54.4 (356)
Prevalent impulsivity dimensions, % (*n*)	
Motor	36.0 (128)
Non-planning	12.1 (43)
Attentional impulsiveness	52.0 (185)

**Table 2:** Differences in sociodemographic and clinical characteristics in patients with and without trait-related impulsivity.

	NIG (*n* = 298)	IG (*n* = 356)
Age (M ± DS)	48.9 ± 14.1	45.2 ± 13.7
Gender, M, % (*n*)	38.9 (116)	43.8 (156)
Living situation, with partner yes % (*n*)	45.3 (135)	33.4 (119)
Years of education (M ± DS)	13.3 ± 3.7	13.3 ± 3.5
Employed, yes, % (*n*)	55.4 (165)	54.2 (193)
Age at onset (M ± DS)	33.5 ± 13.8	29.4 ± 12.1 ***
Diagnosis, % (n)		
Bipolar disorder	37.6 (137)	62.4 (227) ***
Major depression	56.4 (132)	43.6 (102) *
Cyclothymic disorder	50.9 (28)	49.1 (27)
Number of affective episodes (M ± DS)	3.9 ± 4.3	5.1 ± 5.5 ***
Number of hospitalizations (M ± DS)	2.1 ± 1.6	2.5 ± 2.9
Number of involuntary hospitalizations (M ± DS)	1.2 ± 0.5	1.6 ± 1.1 *
Suicide attempts, yes, % (*n*)	6.7 (20)	27.5 (98) ***
Seasonality, yes, % (*n*)	26.2 (78)	37.6 (134) **
Presence of psychotic symptoms during acute phases, yes, % (*n*)	20.8 (62)	37.1 (132) ***

* *p* < 0.01; ** *p* < 0.001; *** *p* < 0.000, NIG: non impulsivity group; IG = impulsivity group.

**Table 3:** Correlations between trait-related impulsivity and prevalent impulsivity dimensions across affective temperaments.

	BIS-11 Total Score ≥ 72	Motor Impulsiveness	Attentional Impulsiveness	Non-Planning Impulsiveness
Cyclothymic temperament	0.156 ***	0.036	−0.017	−0.022
Depressive temperament	−0.108 *	−0.156 *	−0.026	0.164 **
Irritable temperament	0.124 *	0.059	0.029	−0.075
Hyperthymic temperament	−0.036	0.051	0.026	−0.065
Anxious temperament	−0.102 *	0.037	−0.004	−0.032

* *p* < 0.01;** *p* < 0.001; *** *p* < 0.0001.

**Table 4:** Regression analyses exploring the impact of different sociodemographic and clinical characteristics on trait-related impulsivity.

	Model 1			Model 2		
	OR	95% CI		OR	95% CI	
		Lower Bound	Upper Bound		Lower Bound	Upper Bound
Age at onset	0.98 *	0.97	0.99	0.98 *	0.97	0.99
Diagnosis of bipolar disorder	1.16 **	0.62	2.18	1.21	0.63	2.33
Diagnosis of major depression	0.80 *	0.43	1.50	1.04	0.54	1.99
Diagnosis of Cyclothymic disorder	0.91	0.49	1.03	0.89	0.59	1.31
Number of affective episodes	1.00	0.96	1.04	1.00	0.96	1.04
Number of involuntary hospitalizations	1.25	0.88	1.78	1.32	0.92	1.89
Suicide attempts	4.21 ***	2.56	6.92	3.99 ****	2.41	6.63
Seasonality	1.44 *	0.99	2.09	1.43 *	0.98	2.09
Psychotic symptoms during affective episodes	1.56 *	1.03	2.35	1.57 *	1.03	2.41
Cyclothymic temperament	-	-	-	2.12 **	1.29	3.49
Depressive temperament, no	-	-	-	0.87	0.53	1.40
Irritable Temperament, no	-	-	-	3.11 **	1.48	6.52
Anxious Temperament, no	-	-	-	0.84	0.47	1.48

* *p* < 0.05; ** *p* < 0.01;*** *p* < 0.001; **** *p* < 0.0001.

**Table 5:** Regression analyses exploring the impact of different sociodemographic and clinical characteristics on the prevalent impulsiveness dimensions.

	Motor Impulsiveness						Attentional Impulsiveness						Non-planning Impulsiveness					
	Model 1			Model 2			Model 1			Model 2			Model 1			Model 2		
	OR	95% CI		OR	95% CI		OR	95% CI		OR	95% CI		OR	95% CI		OR	95% CI	
		Lower Bound	Upper Bound		Lower Bound	Upper Bound		Lower Bound	Upper Bound		Lower Bound	Upper Bound		Lower Bound	Upper Bound		Lower Bound	Upper Bound
Age at onset	1.01	0.99	1.03	1.01	0.99	1.02	0.99	0.97	1.02	0.99	0.96	1.01	0.99	0.965	1.01	0.99	0.96	1.01
Diagnosis of bipolar disorder	0.55	0.24	1.25	0.52	0.22	1.24	3.42	0.43	26.89	3.16	0.39	25.08	3.42	0.435	26.89	3.16	0.39	25.08
Diagnosis of major depression	0.32 *	0.13	0.76	0.36 *	0.15	0.89	5.33	0.68	41.82	5.41	0.68	42.99	5.33	0.678	41.82	5.41	0.68	42.99
Number of affective episodes	1.07 *	1.02	1.13	1.07 *	1.02	1.13	0.97	0.89	1.06	0.97	0.89	1.06	0.98	0.892	1.06	0.97	0.89	1.06
Number of involuntary hospitalizations	1.06	0.74	1.52	1.08	0.75	1.54	0.89	0.52	1.55	0.89	0.51	1.56	0.89	0.516	1.55	0.89	0.51	1.56
Suicide attempts	0.60	0.35	1.03	0.67	0.38	1.15	0.51	0.23	1.14	0.52	0.23	1.17	0.51	0.226	1.13	0.52	0.23	1.17
Seasonality	1.56	0.96	2.54	1.59	0.97	2.62	1.24	0.61	2.49	1.23	0.61	2.49	1.23	0.615	2.49	1.23	0.61	2.49
Psychotic symptoms during affective episodes	0.70	0.41	1.17	0.72	0.42	1.23	1.41	0.69	2.88	1.46	0.71	3.00	1.41	0.694	2.87	1.46	0.71	3.00
Cyclothymic temperament	-	-	-	0.99	0.45	2.21	-	-	-	1.03	0.33	3.18	-	-	-	1.03	0.33	3.18
Depressive temperament	-	-	-	0.47	0.19	1.12	-	-	-	0.89	0.28	2.87	-	-	-	0.89	0.28	2.87
Irritable Temperament	-	-	-	1.15	0.45	2.89	-	-	-	1.51	0.42	5.45	-	-	-	1.51	0.42	5.45
Anxious Temperament	-	-	-	1.15	0.50	2.62	-	-	-	1.17	0.37	3.72	-	-	-	1.17	0.37	3.72

* *p* < 0.05.

## Data Availability

The data presented in this study are available on request from the corresponding author.
